# Surveillance of Vaccination Coverage Among Adult Populations —United States, 2018

**DOI:** 10.15585/mmwr.ss7003a1

**Published:** 2021-05-14

**Authors:** Peng-Jun Lu, Mei-Chuan Hung, Anup Srivastav, Lisa A. Grohskopf, Miwako Kobayashi, Aaron M. Harris, Kathleen L. Dooling, Lauri E. Markowitz, Alfonso Rodriguez-Lainz, Walter W. Williams

**Affiliations:** ^1^Immunization Services Division, National Center for Immunization and Respiratory Diseases, CDC; ^2^Leidos Inc.; ^3^Influenza Division, National Center for Immunization and Respiratory Diseases, CDC; ^4^Division of Bacterial Diseases, National Center for Immunization and Respiratory Diseases, CDC; ^5^Division of Viral Hepatitis, National Center for HIV/AIDS, Viral Hepatitis, STD, and TB Prevention, CDC; ^6^Division of Viral Diseases, National Center for Immunization and Respiratory Diseases, CDC; ^7^Division of Global Migration and Quarantine, National Center for Emerging and Zoonotic Infectious Diseases, CDC

## Abstract

**Problem/Condition:**

Adults are at risk for illness, hospitalization, disability and, in some cases, death from vaccine-preventable diseases, particularly influenza and pneumococcal disease. CDC recommends vaccinations for adults on the basis of age, health conditions, prior vaccinations, and other considerations. Updated vaccination recommendations from CDC are published annually in the U.S. Adult Immunization Schedule. Despite longstanding recommendations for use of many vaccines, vaccination coverage among U.S. adults remains low.

**Reporting Period:**

August 2017–June 2018 (for influenza vaccination) and January–December 2018 (for pneumococcal, herpes zoster, tetanus and diphtheria [Td]/tetanus toxoid, reduced diphtheria toxoid, and acellular pertussis [Tdap], hepatitis A, hepatitis B, and human papillomavirus [HPV] vaccination).

**Description of System:**

The National Health Interview Survey (NHIS) is a continuous, cross-sectional national household survey of the noninstitutionalized U.S. civilian population. In-person interviews are conducted throughout the year in a probability sample of households, and NHIS data are compiled and released annually. NHIS’s objective is to monitor the health of the U.S. population and provide estimates of health indicators, health care use and access, and health-related behaviors. Adult receipt of influenza, pneumococcal, herpes zoster, Td/Tdap, hepatitis A, hepatitis B, and at least 1 dose of HPV vaccines was assessed. Estimates were derived for a new composite adult vaccination quality measure and by selected demographic and access-to-care characteristics (e.g., age, race/ethnicity, indication for vaccination, travel history [travel to countries where hepatitis infections are endemic], health insurance status, contacts with physicians, nativity, and citizenship). Trends in adult vaccination were assessed during 2010−2018.

**Results:**

Coverage for the adult age-appropriate composite measure was low in all age groups. Racial and ethnic differences in coverage persisted for all vaccinations, with lower coverage for most vaccinations among non-White compared with non-Hispanic White adults. Linear trend tests indicated coverage increased from 2010 to 2018 for most vaccines in this report.

Few adults aged ≥19 years had received all age-appropriate vaccines, including influenza vaccination, regardless of whether inclusion of Tdap (13.5%) or inclusion of any tetanus toxoid–containing vaccine (20.2%) receipt was measured. Coverage among adults for influenza vaccination during the 2017−18 season (46.1%) was similar to the estimate for the 2016−17 season (45.4%), and coverage for pneumococcal (adults aged ≥65 years [69.0%]), herpes zoster (adults aged ≥50 years and aged ≥60 years [24.1% and 34.5%, respectively]), tetanus (adults aged ≥19 years [62.9%]), Tdap (adults aged ≥19 years [31.2%]), hepatitis A (adults aged ≥19 years [11.9%]), and HPV (females aged 19–26 years [52.8%]) vaccination in 2018 were similar to the estimates for 2017.

Hepatitis B vaccination coverage among adults aged ≥19 years and health care personnel (HCP) aged ≥19 years increased 4.2 and 6.7 percentage points to 30.0% and 67.2%, respectively, from 2017. HPV vaccination coverage among males aged 19–26 years increased 5.2 percentage points to 26.3% from the 2017 estimate. Overall, HPV vaccination coverage among females aged 19–26 years did not increase, but coverage among Hispanic females aged 19–26 years increased 10.8 percentage points to 49.6% from the 2017 estimate. Coverage for the following vaccines was lower among adults without health insurance compared with those with health insurance: influenza vaccine (among adults aged ≥19 years, 19–49 years, and 50–64 years), pneumococcal vaccine (among adults aged 19–64 years at increased risk), Td vaccine (among all age groups), Tdap vaccine (among adults aged ≥19 years and 19–64 years), hepatitis A vaccine (among adults aged ≥19 years overall and among travelers aged ≥19 years), hepatitis B vaccine (among adults aged ≥19 years and 19–49 years and among travelers aged ≥19 years), herpes zoster vaccine (among adults aged ≥60 years), and HPV vaccine (among males and females aged 19–26 years). Adults who reported having a usual place for health care generally reported receipt of recommended vaccinations more often than those who did not have such a place, regardless of whether they had health insurance. Vaccination coverage was higher among adults reporting ≥1 physician contact during the preceding year compared with those who had not visited a physician during the preceding year, regardless of whether they had health insurance. Even among adults who had health insurance and ≥10 physician contacts during the preceding year, depending on the vaccine, 20.1%–87.5% reported not having received vaccinations that were recommended either for all persons or for those with specific indications.

Overall, vaccination coverage among U.S.-born adults was significantly higher than that of foreign-born adults, including influenza vaccination (aged ≥19 years), pneumococcal vaccination (all ages), tetanus vaccination (all ages), Tdap vaccination (all ages), hepatitis B vaccination (aged ≥19 years and 19–49 years and travelers aged ≥19 years), herpes zoster vaccination (all ages), and HPV vaccination among females aged 19–26 years. Vaccination coverage also varied by citizenship status and years living in the United States.

**Interpretation:**

NHIS data indicate that many adults remain unprotected against vaccine-preventable diseases. Coverage for the adult age-appropriate composite measures was low in all age groups. Individual adult vaccination coverage remained low as well, but modest gains occurred in vaccination coverage for hepatitis B (among adults aged ≥19 years and HCP aged ≥19 years), and HPV (among males aged 19–26 years and Hispanic females aged 19–26 years). Coverage for other vaccines and groups with Advisory Committee on Immunization Practices vaccination indications did not improve from 2017. Although HPV vaccination coverage among males aged 19–26 years and Hispanic females aged 19–26 years increased, approximately 50% of females aged 19–26 years and 70% of males aged 19–26 years remained unvaccinated. Racial/ethnic vaccination differences persisted for routinely recommended adult vaccines. Having health insurance coverage, having a usual place for health care, and having ≥1 physician contacts during the preceding 12 months were associated with higher vaccination coverage; however, these factors alone were not associated with optimal adult vaccination coverage, and findings indicate missed opportunities to vaccinate remained.

**Public Health Actions:**

Substantial improvement in adult vaccination uptake is needed to reduce the burden of vaccine-preventable diseases. Following the Standards for Adult Immunization Practice (https://www.cdc.gov/vaccines/hcp/adults/for-practice/standards/index.html), all providers should routinely assess adults’ vaccination status at every clinical encounter, strongly recommend appropriate vaccines, either offer needed vaccines or refer their patients to another provider who can administer the needed vaccines, and document vaccinations received by their patients in an immunization information system.

## Introduction

Adults are at risk for illness, hospitalization, disability, and death from vaccine-preventable diseases, particularly influenza and pneumococcal diseases (*1*–*7*). For example, CDC estimates that influenza has resulted in 140,000–810,000 hospitalizations annually since 2010 (*2*). Approximately 50%–70% of these hospitalizations occurred among adults aged ≥65 years, although this age group accounts for only 15% of the U.S. population (*2*). Influenza-associated respiratory and circulatory deaths since 2010 have ranged from a low of 12,000 (during 2011–12) to a high of 61,000 (during 2017–18) (*2*). Despite reduction in disease burden, invasive pneumococcal disease (IPD) remains an important cause of illness and death in the United States, with an estimated 31,000 cases of IPD and 3,590 deaths among persons of all ages in 2017 (*3*). Approximately 90% of these IPD cases and deaths occurred in adults aged ≥18 years (*3*).

CDC recommends vaccinations for adults on the basis of age, health conditions, vaccination history, and other factors (*1*,*4*,*8*) to prevent vaccine-preventable diseases and related outcomes. However, adult vaccination coverage remains low for most routinely recommended vaccines (*4*,*9*) and below *Healthy People 2020* targets (*10*).

In 2018, a composite adult vaccination quality measure was developed to track routinely recommended age-appropriate vaccination among adults, including influenza, pneumococcal, herpes zoster (shingles), and tetanus and diphtheria toxoids (Td) or tetanus toxoid, reduced diphtheria toxoid, and acellular pertussis (Tdap) vaccination (*11*). This report summarizes data on vaccination coverage for U.S. adults aged ≥19 years using data from the 2017 and 2018 National Health Interview Survey (NHIS), an ongoing in-person survey of eligible civilian noninstitutionalized adults (*12*–*14*). Vaccination coverage for the composite adult vaccination quality measure and its component vaccines are assessed as well as associations of vaccination coverage for these and other recommended adult vaccines with demographic characteristics of respondents, including access to health care. The estimates provided in this report can be used by public health practitioners, adult vaccination providers, and the general public to better understand factors that contribute to low adult vaccination rates and to implement or optimize strategies and interventions to improve vaccination coverage.

## Methods

To assess vaccination coverage among adults aged ≥19 years for selected vaccines and demographic factors associated with vaccination, CDC analyzed data from the 2018 NHIS; for influenza vaccination coverage, data from the 2017 NHIS (for August–December) also were used for the 2017 component of the 2017–18 influenza season. This report highlights vaccination estimates for a composite adult vaccination quality measure (*11*), including influenza, pneumococcal, herpes zoster, and Td or Tdap and its component vaccines, and coverage data for other vaccines recommended for adults including hepatitis A, hepatitis B, and human papillomavirus (HPV). Data are reported by selected demographic and access-to-care characteristics (e.g., age, race/ethnicity, indication for vaccination, travel history [travel to countries where hepatitis infections are endemic], health insurance status, contacts with physicians, nativity, and citizenship). Proportions were estimated for adults aged ≥19 years who received selected vaccinations during 2010–2018. For vaccines with narrower age indications (e.g., herpes zoster vaccination indicated only for adults aged ≥50 years), proportions were estimated only among age-eligible adults. Estimates of proportions vaccinated were stratified by age group, risk status, health insurance status, having a usual place for health care, number of physician contacts during the preceding 12 months, nativity, number of years living in the United States, and citizenship.

### Data Source and Collection

NHIS is a national, cross-sectional household survey conducted by the U.S. Census Bureau for CDC’s National Center for Health Statistics (*12*–*14*). The survey samples civilian, noninstitutionalized populations living in the United States at the time of the survey. Face-to-face interviews are conducted weekly throughout the year among a probability sample of U.S. households. NHIS provides estimates of health indicators, health care use and access, and health-related behaviors (*12*–*14*). The final sample adult component response rate was 53.0% for the 2017 NHIS with a total size of 26,742 and 53.1% for the 2018 NHIS with a total size of 25,417 (*13*,*14*). Additional information on NHIS methods has been published previously (https://www.cdc.gov/nchs/nhis/methods.htm).

Vaccination status was determined on the basis of a person’s responses to vaccination questions. Questions about receipt of vaccinations recommended for adults are asked of one randomly selected adult within each family in the household and have been described previously (*15*). A summary is provided of questions asked to ascertain whether adults received influenza, pneumococcal, Td, Tdap, hepatitis A, hepatitis B, herpes zoster (shingles), and HPV vaccines and to determine classification as health care personnel (HCP), whether respondents had health insurance coverage, and whether there is a place to which respondents usually go when sick or need advice on their health (Appendix). There were no questions in the 2018 NHIS to ascertain pneumococcal vaccination by type of vaccine (23-valent pneumococcal polysaccharide vaccine [PPSV23] or 13-valent pneumococcal conjugate vaccine [PCV13]). The 2018 NHIS included questions to ascertain herpes zoster vaccination by type of vaccine (zoster vaccine live [ZVL] versus recombinant zoster vaccine [RZV]), number of vaccine doses received, and timing of vaccine receipt (*13*).

The presence of selected conditions that increase the risk for severe illness and complications from influenza or confer increased risk for pneumococcal disease and are defined by the Advisory Committee on Immunization Practices (ACIP) as indications for influenza and pneumococcal vaccination (Supplementary Box 1; https://stacks.cdc.gov/view/cdc/105321) (*5*,*8*,*16*,*17*) was determined by responses to questions in NHIS. For hepatitis A and hepatitis B vaccination, data also were collected on selected respondent characteristics that increase the risk for infection (for hepatitis A, travel to countries where hepatitis A infections have been endemic since 1995 [travelers] or having chronic liver disease and, for hepatitis B, travel to countries where hepatitis B infections have been endemic since 1995 [travelers], having diabetes, or having chronic liver disease).

Vaccination status and demographic and other characteristics (e.g., health conditions, insurance status, and usual source and frequency of health care) are self-reported. Race/ethnicity was categorized as Hispanic or Latino, Black, White, Asian, and “other.” Persons identified as Hispanic or Latino might be of any race. Persons identified as Black, White, Asian, or other race are non-Hispanic. “Other” includes American Indian/Alaska Native persons and persons of multiple races. The five racial/ethnic categories are mutually exclusive. Nativity was categorized as U.S.-born (persons born in one of the 50 states or the District of Columbia) or foreign-born (persons who were not born in the United States).

### Analysis

Noninstitutionalized adults aged ≥19 years with interviews conducted during August 2017–June 2018 (for influenza vaccination) and January–December 2018 (for pneumococcal, herpes zoster, Td, Tdap, hepatitis A, hepatitis B, and HPV vaccination) were included in this analysis. For noninfluenza adult vaccination coverage estimates, the weighted proportion of respondents who reported receiving selected vaccinations was calculated. To better assess influenza vaccination coverage for the 2017–18 influenza season, reported coverage was restricted to persons who were interviewed during August 2017–June 2018 and vaccinated during July 2017–May 2018, using the Kaplan-Meier survival analysis procedure (*15*). Differences were measured as the simple difference between the 2016–17 and 2017–18 influenza seasons. Data for missing months and years of influenza vaccination (3.8%) were imputed using SAS with Hot-Deck approach.

For the composite adult vaccination quality measure, data from the 2018 NHIS were analyzed to determine estimates for a composite vaccination quality measure of vaccination coverage for select vaccines routinely recommended for all adults aged ≥19 years (Td, Tdap, and influenza vaccine) or indicated on the basis of age (herpes zoster and pneumococcal vaccines) and three age groups (aged 19–49 years, aged 50–64 years, and aged ≥65 years) on the basis of the vaccines recommended for that age group. Estimates for composite measures were calculated to include receipt of Tdap vaccine during the preceding 10 years or receipt of any tetanus toxoid–containing vaccine during the preceding 10 years, and both with and without influenza vaccination during the preceding 12 months.

To assess adjusted vaccination coverage and adjusted prevalence ratios for each vaccine, multivariable logistic regression and predicted marginal modeling were used for selected comparisons. Estimates were adjusted for age, sex, race/ethnicity, marital status, education, employment status, poverty level, number of physician contacts during the preceding year, usual source of health care, self-reported health status, nativity, and region of residence. Income-to-poverty ratio variables are included in the NHIS public use data file (https://www.cdc.gov/nchs/nhis/nhis_2018_data_release.htm). Poverty thresholds were defined according to family size using weighted average U.S. Census Bureau poverty thresholds from 2016, the average Consumer Price Index (CPI) from 2016, actual CPI values for January–July 2017, and projected CPI values for August–December 2017 (*13*).

Weighted data were used to produce national vaccination coverage estimates. Point estimates and 95% confidence intervals were calculated using SUDAAN software (Version 11.0.03; Research Triangle Institute) to account for the complex sample design. *T*-tests were used for comparisons between data years and for comparisons of each level of each respondent characteristic to a chosen referent level (e.g., for race/ethnicity, non-Hispanic White was the reference group). For influenza vaccination, tests for linear trend were performed using a weighted linear regression on the season-specific estimates, using season number as the independent variable and the inverse of the estimated variance of the estimated vaccination coverage as the weights. For vaccination with the other vaccines, tests for linear trend were performed with SUDAAN using the VARGEN procedure. The slope of linear trend analysis on vaccination coverage over years assessed indicated the average annual percentage point increase. Statistical significance was defined as p<0.05. Coverage estimates are not reported for small sample size (n < 30) or relative standard error (standard error/estimates) >0.3.

## Results

Coverage for the adult age-appropriate composite measure was low in all age groups. Racial and ethnic differences in coverage persisted for all vaccinations, with lower coverage for most vaccinations among non-White compared with non-Hispanic White adults. Linear trend tests indicated coverage increased from 2010 to 2018 for most vaccines in this report.

The total adult sample was 25,207 persons aged ≥19 years. The total adult sample for influenza coverage estimation was 21,675 persons aged ≥19 years. Detailed information on vaccination coverage estimates for the composite adult vaccination quality measure is summarized and coverage estimates are stratified by selected populations and variables (Supplementary Boxes 2–5). These selected populations and variables include HCP vaccination (Supplementary Box 2; https://stacks.cdc.gov/view/cdc/105472), hepatitis A and hepatitis B vaccination (Supplementary Box 3; https://stacks.cdc.gov/view/cdc/105322), HPV vaccination (Supplementary Box 4; https://stacks.cdc.gov/view/cdc/105323), and differences in vaccination coverage by selected demographic and access-to-care characteristics (e.g., age, race/ethnicity, indication for vaccination, travel history [travel to countries where hepatitis infections are endemic], health insurance status, contacts with physicians, nativity, and citizenship) (Supplementary Box 5; https://stacks.cdc.gov/view/cdc/105324).

### Coverage for Influenza, Pneumococcal, Herpes Zoster, and Td or Tdap Vaccinations in the Age-Stratified Composite Adult Vaccination Quality Measure

#### Influenza Vaccination Coverage

Influenza vaccination coverage for the 2017–18 season among adults aged ≥19 years was 46.1%, similar to the estimate for the 2016–17 season (Table 1). Coverage in the 2017–18 season among White adults aged ≥19 years was higher (49.3%) than that for Blacks (39.0%), Hispanics (37.5%), and adults reporting other or multiple race (41.4%). Influenza vaccination coverage among adults aged ≥19 years with high-risk conditions was 61.0% during the 2017–18 season, similar to the estimate for the 2016–17 season. Among adults aged ≥19 years with high-risk conditions, coverage was higher among White adults (62.7%) than Black (53.4%) and Hispanic adults (55.0%) but lower than Asian adults (75.9%). Coverage was higher among adults aged ≥19 years with high-risk conditions (61.0%) compared with those without (40.8%). Overall, influenza vaccination coverage among adults aged ≥19 years increased significantly from 37.2% during the 2009–10 season to 46.1% during the 2017–18 season, with average annual percentage point increases of 1.1% (test for trend; p<0.01) (Figure).

**TABLE 1:** Estimated proportion of adults aged ≥19 years who received influenza vaccination, by race/ethnicity* and high-risk status^†^ — National Health Interview Survey, United States, 2017–18 season

Characteristic	Sample size	% (95% CI)	Simple difference from 2016–17
**Overall** ^§^	**21,675**	**46.1 (45.0–47.3)**	**0.7**
**Race/Ethnicity**			
White	14,917	49.3 (48.2–50.5)	1.1
Black	2,322	39.0 (35.5**–**42.7)^¶^	0.5
Hispanic	2,757	37.5 (34.5**–**40.8)^¶^	0.5
Asian	1,082	50.7 (46.4**–**55.2)	0.5
Other	597	41.4 (35.5**–**47.9)^¶^	−6.1
**High-risk status**			
**Adults with high-risk conditions**	**6,260**	61.0 (59.2–62.9)**	**1.3**
White	4,554	62.7 (60.6**–**64.8)**	1.3
Black	683	53.4 (48.0**–**59.1)^¶,^**	−1.5
Hispanic	611	55.0 (48.5**–**61.7)^¶,^**	3.0
Asian	193	75.9 (64.7**–**85.7)^¶,^**	6.1
Other	219	52.4 (42.3**–**63.3)**	−4.1
**Adults without high-risk conditions**	**15,368**	**40.8 (39.5–42.1)**	**0.4**
White	10,329	43.8 (42.5**–**45.2)	0.7
Black	1,636	34.7 (30.7**–**39.1)^¶^	2.5
Hispanic	2,141	33.3 (30.0**–**37.0)^¶^	−0.1
Asian	885	45.2 (40.3**–**50.5)	−0.6
Other	377	35.7 (28.4**–**44.3)^¶^	−7.8

**Abbreviation:** CI = confidence interval.

* Persons identified as White, Black, Asian, or other race are non-Hispanic. Persons identified as Hispanic might be of any race. “Other” includes American Indian/Alaska Native persons and persons who identified multiple races. The five racial/ethnic categories are mutually exclusive.

^†^ Adults categorized as being at high risk for influenza-related complications reported one or more of the following: 1) ever being told by a physician that they had diabetes, emphysema, chronic obstructive pulmonary disease, coronary heart disease, angina, heart attack, or another heart condition; 2) receiving a diagnosis of cancer during the preceding 12 months (excluding nonmelanoma skin cancer) or ever being told by a physician that they had lymphoma, leukemia, or blood cancer; 3) being told by a physician that they had chronic bronchitis or weak or failing kidneys during the preceding 12 months; or 4) reporting an asthma episode or attack during the preceding 12 months.

^§^ Respondents were asked if they had received an influenza shot during the preceding 12 months and, if so, in which month and year. Missing month and year were imputed (3.8%), and interviews conducted during August 2017–June 2018 were used to estimate vaccination coverage during July 2017–May 2018 using Kaplan–Meier survival analysis. Differences were measured as the simple difference between the 2016–17 and 2017–18 influenza seasons.

^¶^ p<0.05 by *t*–test for comparisons with non-Hispanic White as the reference.

** p<0.05 by *t*–test for comparisons between those with high-risk conditions and those without high-risk conditions, overall and within each level of race/ethnicity.

**FIGURE:**
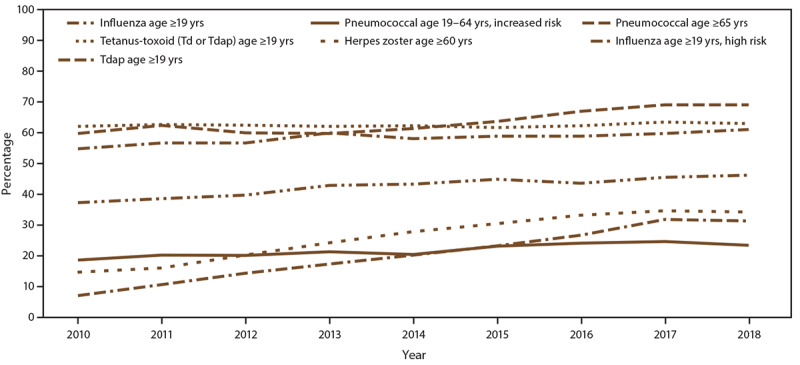
Estimated proportion of adults aged ≥19 years who received selected vaccines, by age group and risk status — National Health Interview Survey, United States, 2010–2018 **NOTE: **An additional table for this figure is available at http://stacks.cdc.gov/view/cdc/105534. **Abbreviations:** Td = tetanus and diphtheria toxoids; Tdap = tetanus toxoid, reduced diphtheria toxoid, and acellular pertussis vaccine.

#### Pneumococcal Vaccination Coverage

Overall pneumococcal vaccination coverage (≥1 dose) (PPSV23 or PCV13) among adults aged 19–64 years at increased risk for pneumococcal disease was 23.3% in 2018, similar to the estimate for 2017 (Table 2). Pneumococcal vaccination coverage for ≥2 doses (vaccine type not ascertained) among adults aged 19–64 years at increased risk was 7.5% in 2018 (Table 2). Overall coverage (≥1 dose) among White adults aged 19–64 years at increased risk was higher (23.6%) compared with Hispanics (18.5%) but did not differ for other racial/ethnic groups. Among adults aged ≥65 years, overall coverage was 69.0%, similar to the estimate for 2017. Among adults aged ≥65 years, coverage for ≥2 doses (vaccine type not ascertained) was 32.3% in 2018. Overall coverage (≥1 dose) among White adults aged ≥65 years (72.6%) was higher compared with Blacks (59.8%), Hispanics (54.3%), and Asians (55.0%) (Table 2). During 2010–2018, pneumococcal vaccination coverage (≥1 dose) among adults aged 19–64 years at increased risk and adults aged ≥65 years ranged from 18.5% through 24.5% and 59.7% through 69.0%, respectively, representing increases in coverage for both age groups (average annual percentage point increase, adults aged 19–64 years at increased risk: 0.7%; average annual percentage point increase, adults aged ≥65 years: 1.3%) (test for trend: p<0.01 for both age groups) (Figure).

**TABLE 2:** Estimated proportion of adults aged ≥19 years who ever received pneumococcal vaccination,* by increased-risk status^†^ and race/ethnicity^§^ **—** National Health Interview Survey, United States, 2018

Characteristic	Sample size	% (95% CI)	Simple difference from 2017
**19–64 yrs at increased risk**			
**Overall**	**5,851**	**23.3 (22.0–24.6)**	**−1.2**
White	4,048	23.6 (22.1–25.2)	−1.3
Black	696	25.7 (21.8–30.0)	3.1
Hispanic	656	18.5 (15.2–22.4)^¶^	−4.5
Asian	192	25.0 (17.3–34.5)	1.7
Other	259	25.8 (19.3–33.5)	−6.5
**≥65 yrs**			
**Overall**	**7,064**	**69.0 (67.5**–**70.4)**	**−0.1**
White	5,434	72.6 (71.1–74.0)	−0.6
Black	706	59.8 (54.7–64.6)^¶^	2.5
Hispanic	509	54.3 (49.2–59.2)^¶^	3.2
Asian	263	55.0 (47.4–62.4)^¶^	−0.6
Other	152	66.1 (55.8–75.1)	4.8
19–64 yrs at increased risk, at least 2 doses**	**5,733**	**7.5 (6.7**–**8.4)**	**NA**
≥65 yrs, at least 2 doses**	**6,669**	**32.3 (30.8**–**33.9)**	**NA**

**Abbreviations:** CI = confidence interval; NA = not available.

* Respondents were asked if they had ever had a pneumonia shot.

^†^ Adults were categorized as being at increased risk for pneumococcal disease if they had ever been told by a doctor or other health professional that they had 1) diabetes, emphysema, chronic obstructive pulmonary disease, coronary heart disease, angina, heart attack, or other heart condition; 2) a diagnosis of cancer during the previous 12 months (excluding nonmelanoma skin cancer); 3) ever been told by a doctor or other health professional that they had lymphoma, leukemia, or blood cancer; 4) been told by a doctor or other health professional that they had chronic bronchitis or weak or failing kidneys during the preceding 12 months; 5) an asthma episode or attack during the preceding 12 months; or 6) were current smokers.

^§^ Persons identified as White, Black, Asian, or other race are non-Hispanic. Persons identified as Hispanic might be of any race. “Other” includes American Indian/Alaska Native persons and persons who identified multiple races. The five racial/ethnic categories are mutually exclusive.

^¶^ p<0.05 by *t*–test for comparisons with non-Hispanic White as the reference.

** Respondents were asked if they had ever had a pneumonia shot and, if yes, were asked how many doses were received. There were no questions in the 2018 National Health Interview Survey to ascertain pneumococcal vaccination by type of vaccine (23-valent pneumococcal polysaccharide vaccine or 13-valent pneumococcal conjugate vaccine).

#### Herpes Zoster Vaccination Coverage

Overall, herpes zoster vaccination coverage among adults aged ≥50 and ≥60 years in 2018 was 24.1% and 34.5%, respectively, similar to the estimates for 2017 (Table 3). White adults aged ≥50 and ≥60 years had higher coverage (28.0% and 38.6%, respectively) compared with Blacks (12.4% and 18.8%, respectively), Hispanics (12.2% and 19.5%, respectively), and Asians (19.6% and 29.1%, respectively) (Table 3). Zoster vaccine live (ZVL) coverage in 2018 was 19.0% among adults aged ≥50 years, 3.7% among adults aged 50–59 years, and 28.0% among adults aged ≥60 years. Coverage for recombinant zoster vaccine (RZV) (≥1 dose) was 2.4% among adults aged ≥50 years, 1.0% among adults aged 50–59 years, and 3.3% among adults aged ≥60 years. RZV coverage (at least 2 doses and received in 2018) was 0.6% among adults aged ≥50 years, 0.8% among adults aged ≥60 years, and 0.8% among adults aged ≥65 years (Table 3). In 2018, ZVL coverage among adults aged ≥60 years was 28.0% (Table 3), a 6.9 percentage point decrease compared with the 2017 estimate (34.9%). During 2010–2018, overall herpes zoster vaccination coverage among adults aged ≥60 years ranged from 14.4% to 34.5%, representing increases in coverage (average annual percentage point increase: 2.8%) (test for trend: p<0.01) (Figure).

**TABLE 3:** Estimated proportion of adults aged ≥50 years who ever received herpes zoster vaccination,* by age and race/ethnicity^†^ — National Health Interview Survey, United States, 2018

Characteristic	Unweighted sample size	% (95% CI)	Vaccinated population size (weighted)	Simple difference from 2017
**≥50 yrs**				
**Overall^§^**	13,486	24.1 (23.1–25.1)	26,687,664	0.2
**Race/Ethnicity**				
White	10,087	28.0 (26.9–29.1)	22,290,713	0.4
Black	1,417	12.4 (10.6–14.5)^¶^	1,398,925	1.1
Hispanic	1,146	12.2 (10.0–14.7)^¶^	1,487,592	−0.1
Asian	509	19.6 (15.8–24.2)^¶^	1,062,651	−4.6
Other	327	19.8 (15.0–25.6)^¶^	447,783	1.3
**≥60 yrs**				
**Overall**	9,401	34.5 (33.2–35.8)	24,356,476	−0.4
**Race/Ethnicity**				
White	7,231	38.6 (37.2–40.1)	20,582,442	−0.7
Black	945	18.8 (16.0–21.9)^¶^	1,204,795	1.7
Hispanic	691	19.5 (15.9–23.8)^¶^	1,288,214	−0.4
Asian	332	29.1 (23.6–35.4)^¶^	893,689	−2.7
Other	202	30.9 (23.3–39.8)	387,336	3.0
**60**–**64 yrs**				
**Total**	**2,310**	**22.5 (20.4–24.8)**	**4,681,477**	**0.1**
White	1,761	25.4 (23.0–28.1)	3,895,942	0.7
Black	241	10.8 (7.0–16.3)^¶^	225,333	−1.9
Hispanic	183	15.3 (8.9–24.9)^¶^	342,136	−2.0
Asian	73	19.7 (12.0–30.7)	161,164	−2.6
Other	52	—^§§^	—	—
**≥65 yrs**				
**Total**	**7,091**	**39.5 (37.9–41.1)**	**19,674,999**	**−0.8**
White	5,470	44.0 (42.3–45.7)	16,686,500	−1.0
Black	704	22.6 (19.2–26.4)^¶^	979,462	3.1
Hispanic	508	21.7 (17.8–26.3)^¶^	946,078	0.3
Asian	259	32.5 (25.8–40.1)^¶^	732,525	−4.3
Other	150	35.5 (26.2–46.1)	330,434	4.2
Zoster vaccine live**				
≥50 yrs	13,125	19.0 (18.1–20.0)	20,583,161	−4.9^¶¶^
50–59 yrs	4,062	3.7 (3.1–4.5)	1,484,353	−2.0^¶¶^
≥60 yrs	9,063	28.0 (26.8–29.3)	19,098,808	−6.9^¶¶^
60–64 yrs	2,268	17.7 (15.8–19.7)	3,606,395	−4.8^¶¶^
≥65 yrs	6,795	32.5 (31.0–34.0)	15,492,413	−7.8^¶¶^
**Recombinant zoster vaccine^††^**				
≥50 yrs	13,211	2.4 (2.1–2.8)	2,647,858	NA
50–59 yrs	4,063	1.0 (0.7–1.5)	415,969	NA
≥60 yrs	9,148	3.3 (2.8–3.7)	2,231,889	NA
60–64 yrs	2,277	2.6 (1.9–3.5)	532,780	NA
≥65 yrs	6,871	3.5 (3.0–4.1)	1,699,109	NA
**Recombinant zoster vaccine, received in 2018, at least 2 doses**				
≥50 yrs	13,164	0.6 (0.5–0.8)	653,911	NA
50–59 yrs	4,060	—	—	NA
≥60 yrs	9,104	0.8 (0.6–1.1)	562,499	NA
60–64 yrs	2,271	—	—	NA
≥65 yrs	6,833	0.8 (0.6–1.1)	391,474	NA

**Abbreviations:** CI = confidence interval; NA = not available.

* Respondents were asked if they had ever received a herpes zoster vaccine. Persons who answered “did not know” or “refused” for each of the “ever had” questions for any zoster vaccine (2.9%), zoster vaccine live (10.0%), and recombinant zoster vaccine (RZV) (7.6%) were excluded from the analysis.

^†^ In this report, persons identified as White, Black, Asian, or other race are non-Hispanic. Persons identified as Hispanic might be of any race. “Other” includes American Indian/Alaska Native persons and persons who identified multiple races. The five racial/ethnic categories are mutually exclusive.

^§^ Respondents were asked if they had ever received a herpes zoster vaccine. Persons who answered “did not know” or “refused” for “ever had any zoster vaccine” (2.9%) were excluded from the analysis.

^¶^ p<0.05 by *t*–test for comparisons with non-Hispanic White as the reference.

** Respondents were asked if they had ever received a herpes zoster vaccine and, if yes, were asked if they ever had zoster vaccine live (requires 1 dose). Persons who answered “did not know” or “refused” for “ever had zoster vaccine live” (10.0%) were excluded from the analysis.

^††^ Respondents were asked if they had ever received a herpes zoster vaccine. If yes, respondents were asked if they ever had recombinant zoster vaccine (requires 2 doses) and, if yes, how many doses. Persons who answered “did not know” or “refused” for “ever had recombinant zoster vaccine (7.6%)” were excluded from the analysis.

^§§^ Estimate is not reliable because of small sample size (n < 30) or if relative standard error (standard error/estimates) was >0.3.

^¶¶^ p<0.05 by *t-*test for comparisons between 2017 and 2018 season within each level of each characteristic.

#### Tetanus Vaccination Coverage

In 2018, the proportion of adults reporting having received any tetanus toxoid–containing vaccination during the preceding 10 years was 62.9% overall for adults aged ≥19 years, 64.5% for adults aged 19–49 years, 62.8% for adults aged 50–64 years, and 58.9% for adults aged ≥65 years (Table 4). The proportion of adults receiving tetanus vaccination during the preceding 10 years across all age groups did not change compared with the estimates for 2017. Overall, White adults had higher coverage compared with Blacks, Hispanics, Asians, and those who indicated other or multiple race (Table 4). During 2010–2018, tetanus vaccination among adults aged ≥19 years was unchanged at approximately 62.0% (Figure).

**TABLE 4:** Estimated proportion of adults aged ≥19 years who received Tdap or any tetanus vaccination* during the preceding 10 years,^†^ by race/ethnicity^§^ and overall by age group **—** National Health Interview Survey, United States, 2018

Characteristic	Sample size	% (95% CI)	Simple difference from 2017
**Any tetanus vaccination**			
**Overall**	**23,813**	**62.9 (61.8–64.0)**	**−0.5**
White	16,360	68.3 (67.2–69.4)	−0.4
Black	2,649	50.2 (47.7–52.8)^¶^	−0.9
Hispanic	2,933	54.0 (51.5–56.5)^¶^	0.3
Asian	1,174	54.7 (50.6–58.8)^¶^	0.7
Other	697	61.9 (57.1–66.4)^¶^	−6.9
**Age (yrs)**			
19–49	10,739	64.5 (63.1–65.8)	0.7
50–64	6,246	62.8 (61.2–64.5)	−1.9
≥65	6,828	58.9 (57.2–60.5)	−1.9
**Tdap vaccination**			
**Overall**	**15,118**	**31.2 (30.0–32.5)**	**−0.5**
White	10,174	36.7 (35.3–38.2)	0.2
Black	1,791	20.1 (17.9–22.6)^¶^	−0.1
Hispanic	1,911	20.5 (18.2–23.1)^¶^	−0.5
Asian	802	25.6 (22.2–29.4)^¶^	−4.2
Other	440	32.0 (26.2–38.3)	−8.4**
**Age (yrs)**			
19–64	10,850	33.5 (32.1–34.9)	0.1
≥65	4,268	22.2 (20.5–24.0)	−2.2

**Abbreviations:** CI = confidence interval; Td = tetanus and diphtheria toxoids; Tdap = Tetanus toxoid, reduced diphtheria toxoid, and acellular pertussis vaccine.

* Respondents were asked if they had received a tetanus shot during the preceding 10 years. Vaccinated respondents included adults who received Td or Tdap during the preceding 10 years.

^†^ Respondents who reported receiving a tetanus shot during the preceding 10 years were asked if their most recent shot included the pertussis or whooping cough vaccine. Among 25,207 respondents aged ≥19 years, those without a “yes” or “no” classification for tetanus vaccination status during the preceding 10 years (n = 1,394 [5.5%]), those who reported tetanus vaccination in the past 10 years but were not told vaccine type by the provider (n = 6,195 [24.6%]), did not know vaccine type (Td or Tdap) (n = 2,495 [9.9%]), or refused to answer or for whom data were not obtained (n = 5 [0.02%]) were excluded, yielding a sample of 15,118 (60.0% of total) respondents aged ≥19 years for whom Tdap vaccination status could be assessed. In February 2012, the Advisory Committee on Immunization Practices recommended Tdap vaccination for all adults aged ≥19 years, including adults aged ≥65 years.

^§^ Persons identified as White, Black, Asian, or other race are non-Hispanic. Persons identified as Hispanic might be of any race. “Other” includes American Indian/Alaska Native persons and persons who identified multiple races. The five racial/ethnic categories are mutually exclusive.

^¶^ p<0.05 by *t*–test for comparisons with non-Hispanic White as the reference.

** p<0.05 by *t*–test for comparisons between 2018 and 2017 for changes in vaccination differences, “difference in difference” analysis, with White as the reference.

Among adults aged ≥19 years for whom Tdap vaccination could be assessed specifically, overall coverage during the preceding 10 years was 31.2%, similar to the estimate for 2017 (Table 4). Tdap coverage for Black (20.1%), Hispanic (20.5%), and Asian (25.6%) adults aged ≥19 years was lower compared with Whites (36.7%). Tdap coverage was 33.5% among adults aged 19–64 years and 22.2% among adults aged ≥65 years. During 2010–2018, Tdap vaccination coverage increased from 6.9% to 31.2% among adults aged ≥19 years (average annual percentage point increase: 3.2%) (test for trend: p<0.01) (Figure).

### Vaccination Estimates Using an Age-Appropriate Composite Adult Vaccination Quality Measure

In 2018, few adults aged ≥19 years had received all age-appropriate vaccines (including influenza vaccination) included in the composite measure regardless of whether inclusion of Tdap (13.5%) or inclusion of any tetanus toxoid–containing vaccine (20.2%) was measured (Table 5). Coverage for the composite adult vaccination quality measure (with influenza vaccination, any toxoid-containing vaccine) was low in all age groups, ranging from 6.7% among adults aged 50–64 years to 25.7% among adults aged 19–49 years. Adults aged 50–64 years had the lowest composite vaccination coverage (Tdap: 3.9%; any toxoid-containing vaccine: 6.7%). Low coverage with herpes zoster vaccine in this age group (11.5%) was the primary driver of this result.

**TABLE 5:** Vaccination coverage estimates using an age-appropriate composite* adult vaccination quality measure and individual component measures, by age group — National Health Interview Survey, United States, 2018

Characteristic	% (95% CI)			
	≥19 yrs	19–49 yrs	50–64 yrs	≥65 yrs
	(n = 25,207)^†^	(n = 11,318)^†^	(n = 6,592)^†^	(n = 7,297)^†^
**Composite measure**				
**Includes influenza during preceding 12 months**				
Tdap only^§^	13.5 (12.7–14.3)	18.7 (17.4–19.9)	3.9 (3.2–4.8)	11.2 (10.0–12.5)
Td or Tdap^¶^	20.2 (19.4–21.0)	25.7 (24.5–26.9)	6.7 (6.0–7.6)	22.6 (21.2–24.0)
**Does not include influenza during preceding 12 months**				
Tdap only**	24.0 (22.9–25.2)	36.9 (35.2–38.6)	4.8 (4.1–5.7)	12.1 (10.9–13.5)
Td or Tdap^††^	42.3 (41.3–43.3)	64.5 (63.1–65.8)	8.7 (7.9–9.7)	25.4 (23.9–26.8)
**Component measures^§§^**				
Influenza during preceding 12 months	44.7 (43.8–45.6)	34.2 (33.0–35.4)	46.9 (45.3–48.4)	68.8 (67.4–70.1)
Td or Tdap	62.9 (61.8–64.0)	64.5 (63.1–65.8)	62.8 (61.2–64.5)	58.9 (57.2–60.5)
Tdap	31.2 (30.0–32.5)	36.9 (35.2–38.6)	26.0 (24.2–27.9)	22.2 (20.5–24.0)
Herpes zoster^¶¶^	24.1 (23.1–25.1)	—	11.5 (10.5–12.5)	39.5 (37.9–41.1)
Pneumococcal***	69.0 (67.5–70.4)	—	—	69.0 (67.5–70.4)

**NOTE: **An additional table for this figure is available at http://stacks.cdc.gov/view/cdc/105326

**Abbreviations:** CI = confidence interval; Td = tetanus and diphtheria toxoids vaccine; Tdap = tetanus toxoid, reduced diphtheria toxoid, and acellular pertussis vaccine.

* Estimates using data from the 2018 National Health Interview Survey for age-based composite adult vaccination quality measure for tetanus toxoid-containing, pneumococcal, herpes zoster, and influenza vaccines. Influenza vaccination was measured as receipt during the preceding 12 months, in contrast to Table 1, where influenza vaccination coverage for July 2017–May 2018 was estimated using Kaplan–Meier survival analysis. Td/Tdap vaccination was measured as receipt during the preceding 10 years. Pneumococcal and herpes zoster vaccination were measured as ever receiving at least one dose of either kind of vaccine. Estimates for the composite measures (**Source:** Shen AK, Williams WW, O'Halloran AC, et al. Promoting adult immunization using population-based data for a composite measure. Am J Prev Med 2018;55:517–23) were calculated to include Tdap vaccine during the preceding 10 years (Method 1) or any tetanus-toxoid containing vaccine during the preceding 10 years (Method 2), and both with and without influenza vaccination during the preceding 12 months. Using Method 1, percent of respondents excluded in vaccination coverage estimation including influenza vaccination was 40.7% overall, ranging from 38.4% in adults aged 19–49 years to 43.1% in adults aged ≥65 years; percent of respondents excluded in vaccination coverage estimation excluding influenza vaccination was 40.6% overall, ranging from 38.2% in adults aged 19–49 years to 43.1% in adults aged ≥65 years.

^†^ Total unweighted sample size for the overall age group. Denominators for each point estimate vary because persons who did not answer vaccination questions were excluded from analysis.

^§^ A composite estimate of overall vaccination coverage among adults aged ≥19 years who received the selected vaccines recommended for their age group: for adults aged 19–49 years, influenza and Tdap vaccines; for adults aged 50–64 years, influenza, Tdap, and herpes zoster vaccines; for adults aged ≥65 years, influenza, Tdap, herpes zoster, and pneumococcal vaccines. Estimates for each age group include adults who have received all of the selected vaccines for that specific age group.

^¶^ A composite estimate of overall vaccination coverage among adults aged ≥19 years who received the selected vaccines recommended for their age group: for adults aged 19–49 years, influenza AND Td or Tdap vaccines; for adults aged 50–64 years, influenza, Td or Tdap, and herpes zoster vaccines; for adults aged ≥65 years, influenza, Td or Tdap, herpes zoster, and pneumococcal vaccines. Estimates for each age group include adults who have received all of the selected vaccines for that specific age group.

** A composite estimate of overall vaccination coverage among adults aged ≥19 years who received the selected vaccines recommended for their age group: for adults aged 19–49 years, Tdap vaccines; for adults aged 50–64 years, Tdap and herpes zoster vaccines; for adults aged ≥65 years, Tdap, herpes zoster, and pneumococcal vaccines. Estimates for each age group include adults who have received all of the selected vaccines for that specific age group.

^††^ A composite estimate of overall vaccination coverage among adults aged ≥19 years who received the selected vaccines recommended for their age group: for adults aged 19–49 years, Td or Tdap vaccines; for adults aged 50–64 years, Td or Tdap and herpes zoster vaccines; for adults aged ≥65 years, Td or Tdap, herpes zoster, and pneumococcal vaccines. Estimates for each age group include adults who have received all of the selected vaccines for that specific age group.

^§§^ Definitions of component measures for Td/Tdap, herpes zoster, and pneumococcal vaccines appear in the footnotes of Tables 2–4. For influenza, respondents were asked if they received an influenza shot or nasal spray during the preceding 12 months.

^¶¶^ Herpes zoster vaccination coverage among adults aged ≥50 years.

*** Pneumococcal vaccination coverage among adults aged ≥65 years.

### Additional Vaccination Coverage Estimates Not Included in the Composite Adult Vaccination Quality Measure

#### Vaccination Coverage Among HCP Aged >19 Years for Selected Vaccines

##### Influenza, Hepatitis B, and Tdap Vaccination Among HCP

Overall in 2018, influenza and Tdap vaccination coverage among HCP aged ≥19 years (71.8% and 55.8%, respectively) were similar to the 2017 estimates. Hepatitis B vaccination coverage increased among HCP aged ≥19 years (67.2%) compared with the 2017 estimate (60.5%). Among all HCP, White HCP had higher Tdap (60.9%) and hepatitis B coverage (70.9%) compared with Black HCP (37.9% and 56.3%, respectively) and Hispanic HCP (46.7% and 57.2%, respectively) (Supplementary Box 2, Table 1; https://stacks.cdc.gov/view/cdc/105472). During 2010–2018, influenza and Tdap vaccination coverage increased among HCP aged ≥19 years, and hepatitis B vaccination coverage remained stable (Supplementary Box 2, Figure; https://stacks.cdc.gov/view/cdc/105472).

##### Influenza, Hepatitis B, and Tdap Vaccination Among HCP with or Without Direct Patient Care

Overall in 2018, influenza and Tdap vaccination coverage among HCP aged ≥19 years with (72.6% and 60.2%, respectively) or without direct patient care (70.5% and 46.6%, respectively) were similar to the 2017 estimates. Hepatitis B vaccination coverage increased 5.5 percentage points to 75.3% among HCP aged ≥19 years with direct patient care, compared with the estimate for 2017. Hepatitis B vaccination coverage increased 8.1 percentage points to 50.9% among HCP aged ≥19 years without direct patient care, compared with the estimate for 2017. Among HCP with direct patient care, influenza coverage among White HCP (72.3%) was similar compared with that for Black (75.0%) and Hispanic HCP (70.3%). White HCP aged ≥19 years with direct patient care responsibilities had higher hepatitis B coverage (82.3%) compared with Black (58.1%) and Hispanic HCP (62.6%) (Supplementary Box 2, Table 2; https://stacks.cdc.gov/view/cdc/105472).

##### Proportion of Adults Who Received Tdap Among Those Reporting Tetanus Vaccination by HCP Status

Among adults aged ≥19 years, 42.6% reported that they knew which type of tetanus vaccine they received, 40.6% reported that they were not informed of the vaccination type, and 16.8% could not recall the type of tetanus vaccination received. Among those who reported that they knew which type tetanus vaccine they received, 74.4% reported receiving Tdap. HCP reported receipt of Tdap more often (83.0%) than did non-HCP (72.7%) (Supplementary Box 2, Table 3; https://stacks.cdc.gov/view/cdc/105472).

#### Hepatitis A and Hepatitis B Vaccination

##### Hepatitis A Vaccination

In 2018, among adults aged ≥19 years, hepatitis A vaccination coverage (≥2 doses) was similar to the estimates for 2017, overall (11.9%), among travelers (18.9%), and among adults with chronic liver conditions (15.8%). Hepatitis A vaccination coverage was higher among travelers aged ≥19 years (18.9%) than nontravelers (7.4%). Among adults aged 19–49 years, Whites had higher hepatitis A vaccination coverage (18.2%) than Blacks (12.8%) but lower than Asians (24.1%) (Supplementary Box 3, Table 1; https://stacks.cdc.gov/view/cdc/105322). During 2010–2018, among all adults aged ≥19 years, hepatitis A vaccination coverage increased (range: 8.1%–11.9%; test for trend: p<0.01). Coverage also increased during 2010–2018 among travelers (range: 14.6%–18.9%; test for trend: p<0.01), and among nontravelers (range: 5.1%–7.4%; test for trend: p<0.01) but remained stable among persons with chronic liver conditions (range: 8.6%–20.8%; test for trend: p>0.05) (Supplementary Box 3, Figure; https://stacks.cdc.gov/view/cdc/105322).

##### Hepatitis B Vaccination

Hepatitis B vaccination coverage (≥3 doses) in 2018 increased among adults aged ≥19 years overall (30.0%), among travelers (38.9%), and among nontravelers (24.2%), compared with the estimates for 2017. Hepatitis B vaccination coverage in 2018 increased among adults aged 19–59 years with diabetes (33.0%) compared with the estimates for 2017. Hepatitis B vaccination coverage was higher among travelers (38.9%) than nontravelers aged ≥19 years (24.2%) and higher among adults with diabetes aged 19–59 years (33.0%) than adults with diabetes aged ≥60 years (15.3%). Among adults aged 19–49 years, Whites had higher hepatitis B vaccination coverage (43.6%) compared with Blacks (35.4%) and Hispanics (33.1%) (Supplementary Box 3, Table 2; https://stacks.cdc.gov/view/cdc/105322). During 2010–2018 among all adults aged ≥19 years, hepatitis B vaccination coverage increased (range: 24.5%–30.0%; test for trend: p = 0.01) but remained stable among travelers aged ≥19 years, nontravelers aged ≥19 years, and adults aged ≥19 years with chronic liver conditions (Supplementary Box 3, Figure; https://stacks.cdc.gov/view/cdc/105322).

#### HPV Vaccination

In 2018 among females, HPV vaccination coverage (reported receipt of at least 1 dose of HPV vaccine) by age group was 52.8% (19–26 years), 53.3% (19–21 years), and 52.5% (22–26 years). Among males aged 19–21 years, HPV vaccination coverage was 34.4%. HPV vaccination coverage in 2018 for females by age group (19–26, 19–21, and 22–26 years) and for males aged 19–21 years was similar to the estimates for 2017 (Supplementary Box 4, Table 1; https://stacks.cdc.gov/view/cdc/105323). In 2018, HPV vaccination coverage among males aged 19–26 years (26.3%) and 22–26 years (21.8%) was higher than 2017 estimates (Supplementary Box 4, Table 1; https://stacks.cdc.gov/view/cdc/105323). Most females (79.9%) and males (87.4%) reported receiving the first dose of HPV vaccine at age ≥13 years (Supplementary Box 4, Table 2; https://stacks.cdc.gov/view/cdc/105323). HPV vaccination increased from 20.7% in 2010 to 52.8% in 2018 among females aged 19–26 years and from 2.1% in 2011 to 26.3% in 2018 among males aged 19–26 years (test for trend: p<0.01 for both groups) (Supplementary Box 4, Figure; https://stacks.cdc.gov/view/cdc/105323).

### Adult Vaccination Coverage by Race/Ethnicity, Age, Access-to-Care Characteristics, Nativity, Years Living in the United States, and Citizenship

#### Racial and Ethnic Vaccination Differences

Compared with 2017, racial/ethnic differences in vaccination coverage persisted for all seven vaccines in this report. With Whites as the reference group, there were differences in vaccination coverage for 48 of the 66 comparisons by vaccine and age/target groups (not including comparisons of the “other” racial/ethnic group) (Supplementary Box 5, Table 1; https://stacks.cdc.gov/view/cdc/105324). During 2010–2018, vaccination differences between Whites, Blacks, Hispanics, Asians, and persons reporting other race increased for select vaccines and age groups (tetanus vaccination [Td or Tdap; adults aged ≥19 years and 19–49 years], Tdap [adults aged ≥19 years and 19–64 years], hepatitis A [adults aged 19–49 years], hepatitis B [adults aged 19–49 years and HCP aged ≥19 years], and herpes zoster vaccination [adults aged ≥60 years and ≥65 years]) (Supplementary Box 5, Table 2; https://stacks.cdc.gov/view/cdc/105324). Vaccination differences between Whites, Blacks, Hispanics, and persons reporting other race for the other vaccines and age groups did not change during this period. Vaccination differences by race/ethnicity for influenza, tetanus, and herpes zoster vaccination were observed for additional age groups (Supplementary Box 5, Tables 3–5; https://stacks.cdc.gov/view/cdc/105324).

#### Association of Health Insurance Status with Adult Vaccination Coverage

Overall, vaccination coverage was generally lower among adults without health insurance compared with those with health insurance. Adult vaccination coverage differed by the type of health insurance. With the exception of influenza vaccination and pneumococcal vaccination, vaccination coverage was higher among adults with private health insurance compared with those reporting public health insurance (Supplementary Box 5, Table 6; https://stacks.cdc.gov/view/cdc/105324). After adjustment for selected demographic, access-to-care characteristics, and other variables, adults without health insurance were significantly less likely than those with health insurance to be vaccinated (Supplementary Box 5, Table 7; https://stacks.cdc.gov/view/cdc/105324).

#### Association of Having a Usual Place for Health Care with Adult Vaccination Coverage

Generally, adults with a usual place for health care were more likely to report having received recommended vaccinations than those who did not have a usual place for health care, regardless of whether they had health insurance (Supplementary Box 5, Table 8; https://stacks.cdc.gov/view/cdc/105324).

#### Adult Vaccination Coverage by Number of Physician Contacts

Generally, vaccination coverage was higher among those reporting having had ≥1 physician contacts during the preceding year compared with those who had not visited a physician during the preceding year, regardless of whether they had health insurance. In addition, vaccination coverage generally increased as the number of physician contacts increased. Among adults who had health insurance and ≥10 physician contacts during the preceding year, 20.1%–87.5% reported not having received a vaccine or vaccine series that was recommended either for all persons or for those with some specific indication (Supplementary Box 5, Table 9; https://stacks.cdc.gov/view/cdc/105324).

#### Association of Respondent Age with Adult Vaccination Coverage

Influenza vaccination coverage among adults aged ≥65 years was higher (72.2%) than coverage among adults aged 19–49 years (34.8%) and 50–64 years (48.1%), and pneumococcal vaccination coverage among adults aged ≥65 years was higher (69.0%) than coverage among adults aged 19–64 years (23.3%). However, overall tetanus vaccination (Td or Tdap) coverage among adults aged ≥65 years (58.9%) was lower compared with coverage among adults aged 19–49 years (64.5%) and 50–64 years (62.8%), and Tdap vaccination coverage among adults aged ≥65 years was lower (22.2%) than coverage among adults aged 19–64 years (33.5%). Hepatitis B vaccination coverage among adults aged ≥60 years with diabetes was lower (15.3%) compared with coverage among adults aged 19–59 years with diabetes (33.0%). Herpes zoster coverage among adults ≥65 years was higher (39.5%) compared with coverage among adults aged 60–64 years (22.5%) (Tables 2 and 3) (Supplementary Box 3, Table 2; https://stacks.cdc.gov/view/cdc/105322) (Supplementary Box 5, Tables 3, 4, 6, 8, and 9; https://stacks.cdc.gov/view/cdc/105324).

#### Adult Vaccination Coverage by Nativity, Years Living in the United States, and Citizenship

Overall, vaccination coverage among U.S.-born adults was significantly higher than that seen in foreign-born adults including influenza vaccination (aged ≥19 years: 47.0% versus 42.2%), pneumococcal vaccination (aged 19–64 years at increased risk: 24.2% versus 17.4%; and aged ≥65 years: 72.1% versus 51.3%), tetanus vaccination (aged ≥19 years: 65.8% versus 51.1%; aged 19–49 years: 67.2% versus 54.0%; aged 50–64 years: 66.7% versus 47.5%; and aged ≥65 years: 61.0% versus 46.6%), Tdap vaccination (aged ≥19 years: 34.4% versus 18.1%; aged 19–64 years: 37.2% versus 19.5%; and aged ≥65 years: 24.2% versus 11.2%), hepatitis B vaccination (aged ≥19 years: 30.7% versus 27.2%; 19–49 years: 42.4% versus 32.1%; and travelers aged ≥19 years: 40.6% versus 34.2%), herpes zoster vaccination (aged ≥60 years: 36.8% versus 21.3%; aged 60–64 years: 24.6% versus 11.6%; and aged ≥65 years: 41.9% versus 25.6%), and HPV vaccination among females aged 19–26 years (54.7% versus 39.5%). Compared with U.S.-born adults, there were large gaps (≥14 percentage points) in vaccination coverage among foreign-born adults for pneumococcal vaccination (adults aged ≥65 years), tetanus vaccination (Td or Tdap) (adults aged ≥19 years, 50–64 years, and ≥65 years), Tdap vaccination (adults aged ≥19 years and 19–64 years), herpes zoster vaccination (adults aged ≥60 years and ≥65 years), and HPV vaccination (females aged 19–26 years). Vaccination status among foreign-born adults varied by time living in the United States and citizenship (Supplementary Box 5, Table 10; https://stacks.cdc.gov/view/cdc/105324).

## Discussion

NHIS data indicate that many adults in the United States remained unprotected against vaccine-preventable diseases in 2018. Adult vaccination coverage remained similar to that in 2017 for most vaccines, with modest increases observed only for hepatitis B vaccination and HPV vaccination (males aged 19–26 years and Hispanic females aged 19–26 years). Having health insurance coverage, having a usual place for health care, and having ≥1 physician contact during the preceding year were associated with higher vaccination coverage. Vaccination coverage estimates for three of the four vaccines in this report that are included in *Healthy People 2020* (influenza, pneumococcal, and hepatitis B [for HCP] vaccines) were below the respective target levels, even among insured adults and adults with multiple health care visits during the preceding year (*10*). Herpes zoster vaccination coverage in 2018 was 4.5 percentage points above the *Healthy People 2020* target of 30% (*10*). Racial and ethnic differences in vaccination coverage persisted for all vaccinations with lower coverage generally for most vaccinations among non-White and Hispanics compared with non-Hispanic White adults. Depending on the vaccine, 20.1%–87.5% reported not having received vaccinations among adults who had health insurance and ≥10 physician contacts during the preceding year, indicating multiple missed opportunities for vaccination and the need to increase routine assessment of adult vaccination needs and vaccination with recommended vaccines.

### Composite Adult Vaccination Quality Measure

Coverage for the age-appropriate composite measures was low in all age groups. The composite adult vaccination quality measure presented in this report was adopted by the Indian Health Service and added to the Healthcare Effectiveness Data and Information Set (HEDIS) by the National Committee for Quality Assurance (NCQA) for first-year reporting beginning in 2018 (*18*). HEDIS is a set of national performance measures used to compare health plans and drive improvement in important facets of health care delivery. NCQA added the Adult Immunization Status measure to the HEDIS Health Plan Set to assess routine vaccination for select vaccines. The measure includes four rates assessing receipt of influenza, Td/Tdap, herpes zoster, and pneumococcal vaccination for adults aged ≥19 years and a composite rate to provide a summary of performance across these different vaccines. The composite rate assessed the total number of vaccines that were received across a health plan’s member population per clinical guidelines (i.e., the sum of the individual vaccines administered divided by the sum of the individual vaccines required). The measure was specified for the HEDIS Electronic Clinical Data Systems reporting method. Data sources included administrative claims, electronic medical records, registries, case management systems, and health information exchanges.

The vaccination coverage estimates for the composite adult vaccination quality measure presented in this report derived from self-report of vaccination status will differ from those generated by the NCQA, which are based on vaccination records from electronic clinical data systems for members enrolled in participating health plans. In addition, CDC and NCQA use different approaches for calculating coverage estimates. The CDC analytic approach uses persons as the unit of analysis, where estimates for each age group represent the proportion of adults who reported receipt of all the vaccines routinely recommended for that age group. The composite numerator for CDC estimates includes only those persons who reported receiving all the recommended vaccines (a unit of person); the composite denominator for estimates includes all the persons with indications for vaccination on the basis of the recommended vaccines for that specific age group (a unit of person, each person counted once) (Supplementary Table, https://stacks.cdc.gov/view/cdc/105325). The NCQA analytic approach (*19*) uses recommended vaccines as the unit of analysis; specifically, the number of vaccinations administered or contraindicated (numerator) out of the possible number of vaccinations needed by plan members according to ACIP recommendations for the age group (denominator) (i.e., the percentage of the total recommended number of vaccinations, per the guidelines for that age, that were administered as indicated [i.e., the sum of the individual vaccines administered divided by the sum of the individual vaccines required]). Also, in contrast to the CDC approach, NCQA uses actual vaccination data from the participating health plans (commercial, Medicare, and Medicaid) to generate estimates, different exclusion criteria for analyses than CDC, different measurement periods for ascertaining influenza and Td/Tdap vaccination status, and different criteria for herpes zoster vaccination (e.g., the criteria are vaccine-type specific with the recombinant zoster vaccine criterium requiring series completion to be counted). In addition, the NCQA criterion for pneumococcal vaccination of plan members aged ≥66 years at the start of the measurement period was based on the previous “series completion” ACIP recommendations in effect during 2018 (i.e., receipt of both PCV13 and PPSV23 in series with the recommended interval based on whether the recipient was pneumococcal vaccine-naïve or had previously received PPSV23) (Supplementary Table, https://stacks.cdc.gov/view/cdc/105325) (*20*). For the comparison estimates in this report, recommended vaccines were adapted as the unit of analysis. The composite numerator of the adapted NCQA approach indicates whether the vaccination was administered (a unit of recommended vaccinations received). The composite denominator indicates the number of recommended vaccinations for persons based on their age (a unit of recommended vaccinations). For the adapted NCQA approach, influenza vaccination was measured as receipt during the preceding 12 months, Tdap was measured as receipt during the preceding 10 years, and herpes zoster vaccines and pneumococcal vaccine were measured as having ever received these vaccinations. For actual NCQA estimates: 1) influenza vaccination was measured as receipt on or between July 1 of the year before the measurement period and June 30 of the measurement period; 2) Td/Tdap vaccination was measured as receipt of at least one Td or Tdap vaccine between 9 years before the start of the measurement period and the end of the measurement period; 3) persons received at least 1 dose of the herpes zoster live vaccine or 2 doses of the herpes zoster recombinant vaccine (at least 28 days apart) anytime on or after the person’s 50th birthday; and 4) persons were administered both the 13-valent pneumococcal conjugate vaccine and the 23-valent pneumococcal polysaccharide vaccine at least 12 months apart, with the first occurrence after the age of 60 years.

NCQA revisited the usefulness of the composite rate during a re-evaluation of the measure in 2020. Stakeholder feedback included concerns about the usability of the composite rate as constructed, particularly with combining vaccines recommended for younger versus older adults into a composite. Thus, NCQA removed the composite rate from the Adult Immunization Status measure in 2020. The four individual vaccine rates for influenza, Td/Tdap, herpes zoster, and pneumococcal vaccination will continue to be reported.

Composite performance measures, which combine multiple individual (“component”) quality measures, provide a useful way to examine overall health system performance in implementing standards of care as well as a reminder and an incentive for implementing these standards by providers (*11*). The U.S. Department of Health and Human Services has proposed a developmental *Healthy People 2030* (HP2030) composite adult vaccination quality measure as a new objective to assess overall adult vaccination performance (*21*). This developmental measure targets increasing the proportion of adults age ≥19 years who receive recommended age-appropriate vaccines. This objective is a high-priority public health issue with evidence-based interventions; however, reliable baseline data are required before it can become a core HP2030 objective. The composite adult vaccination quality measure estimates in this report indicate that, despite variable coverage with individual recommended vaccines, few adults in any age group were fully vaccinated according to ACIP recommendations. Presenting both composite and component measures allows assessment of overall performance and targeted interventions for improvement.

### Influenza Vaccination

Since the 2010–11 influenza season, ACIP has recommended annual influenza vaccination for all persons aged ≥6 months (*22*). By the 2017–18 season (seven seasons after annual influenza vaccination was recommended for all adults), vaccination coverage among adults aged ≥19 years was 46.1%, with an average annual 1.1 percentage point increase from the 2009–10 through the 2017–18 seasons. However, by the 2017–18 season, approximately 50% of adults had not received influenza vaccine, and coverage was well below the *Healthy People 2020* target of 70% (*10*). In addition, coverage among adults aged ≥19 years with high-risk conditions remained low (61.0% in the 2017–18 season). Even after its universal influenza vaccination recommendation, ACIP continued to emphasize that persons with high-risk conditions should be a focus of vaccination efforts (*22*). Persons with underlying health conditions might not consider themselves as high risk, limiting the effectiveness of targeted messages. Many persons with high-risk conditions see subspecialists, who are less likely to recommend influenza vaccination than general practitioners (*23*).

Vaccination of HCP is an important component of influenza prevention programs in the United States (*24*). Vaccination of HCP could reduce transmission of influenza in health care settings, staff illness and absenteeism, and influenza-related morbidity and mortality (*24*). Despite the availability of safe and effective influenza vaccines (*25*,*26*), influenza vaccination coverage among HCP remains suboptimal (*4*,*15*,*27*–*30*). By the 2017–18 season, vaccination coverage among HCP overall (71.8%) and among HCP with and without direct patient care (72.6% and 70.5%, respectively) remained far below the *Healthy People 2020* target for HCP of 90% (*10*).

Previous studies of influenza illnesses and hospitalizations that could be averted by vaccination have indicated that higher vaccination rates could prevent a substantial number of influenza cases and hospitalizations (*31*). For example, one study indicated that a 5% influenza vaccination coverage increase would result in 785,000 fewer illnesses (56% among those aged 18–64 years) and 11,000 fewer hospitalizations (*31*). More effort is needed to reach the *Healthy People 2020* and *2030* targets to benefit fully from influenza vaccination (*10*,*32*). Ensuring that all persons who visit a health care provider during the influenza season receive a vaccination recommendation and offer from their provider and use of immunization information systems could increase influenza vaccination rates (*33*,*34*). Employers and health care administrators also should implement evidence-based interventions to increase influenza vaccination coverage among HCP, including on-site vaccination at no or low cost to HCP (*30*).

### Pneumococcal Vaccination

The overall pneumococcal vaccination estimates in this report include respondents who received PCV13, PPSV23, or both. Respondents indicating receipt of ≥2 doses of pneumococcal vaccine include adults who are recommended to receive 1 dose of PPSV23 only, or a dose of PCV13 and up to 2 doses of PPSV23 (*17*,*35*). Since 1997, ACIP has recommended PPSV23 vaccination of all adults aged ≥65 years and younger adults with chronic or immunocompromising medical conditions (*35*). In 2012, ACIP recommended PCV13 to adults aged 19–64 years at increased risk and, in 2014, ACIP recommended routine use of PCV13 in series with PPSV23 for all adults aged ≥65 years (*17*,*36*). At that time, ACIP recognized that there would be a need to reevaluate this recommendation because it was anticipated that PCV13 use in children would continue to reduce disease burden among adults through reduced carriage and transmission of vaccine serotypes from vaccinated children (i.e., PCV13 indirect effects). On June 26, 2019, after having reviewed the evidence accrued during the preceding 3 years (*37*), ACIP voted to remove the recommendation for routine PCV13 use among adults aged ≥65 years and to recommend administration of PCV13 based on shared clinical decision-making for adults aged ≥65 years who do not have an immunocompromising condition, cerebrospinal fluid leak, or cochlear implant, and who have not previously received PCV13. All adults aged ≥65 years should continue to receive 1 dose of PPSV23 (*36*). Recommendations and guidance and implementation considerations for recommendations on shared clinical decision-making are available (*37*,*38*).

Pneumococcal vaccination of persons aged 19–64 years at increased risk increased during 2010–2018 but remains well below the *Healthy People 2020* target of 60% (*10*). Millions of adults in the United States have conditions placing them at increased risk for complications of pneumococcal disease or other vaccine-preventable infections (*39*,*40*). Adults with certain chronic and immunocompromising health conditions are at substantially increased risk for IPD compared with adults without these conditions, with disease rates up to 33 times higher in some immunocompromised adults (*41*). In this report, only one fourth of adults aged 19–64 years at increased risk reported ever receiving a dose of pneumococcal vaccine, leaving approximately 70% of adults at increased risk unprotected. Pneumococcal vaccination of adults aged ≥65 years increased during 2010–2018; however, coverage remains well below the *Healthy People 2020* target of 90% (*10*). Achieving higher pneumococcal vaccination levels can reduce morbidity and mortality related to pneumococcal disease.

### Herpes Zoster Vaccination

Overall, in 2018, herpes zoster vaccination coverage among adults aged 50–59 years was 5.8%, similar to the estimate for 2017. ZVL was licensed by the U.S. Food and Drug Administration (FDA) for adults aged ≥50 years, but not recommended by ACIP for adults aged 50–59 years. The ACIP recommendation was driven by concerns about waning immunity of ZVL in vaccine recipients aged 50–59 years combined with increasing risk for herpes zoster with age and cost-effectiveness analyses (*42*). In October 2017, ACIP recommended the recent FDA-approved RZV for use in immunocompetent adults aged ≥50 years, revaccination of those who previously received ZVL, and preferential use of RZV over ZVL because of its higher and more long-lasting efficacy (*43*). The differences between FDA’s ZVL licensing and ACIP recommendations for ZVL use likely influenced the usage patterns of ZVL before widespread distribution of RZV. The limited use of ZVL in persons aged 50–59 years likely reflects use of an FDA-approved vaccine among some vaccination providers and individual clinical decision-making with their patients, illustrating the strong influence of ACIP recommendations on national vaccination practices.

ZVL coverage among adults aged ≥60 years was 34.9% in 2017 (*9*) and 28% among the same age group in 2018. Even if no ZVL had been administered in 2018, that might be insufficient to explain the decreased coverage compared with 2017. This observed decrease in coverage might reflect the effect of the change in herpes zoster vaccination recommendations in October 2017 and the questions asked in the 2018 NHIS to ascertain type of herpes zoster vaccine received. In 2017, respondents were asked if they had ever received a shingles vaccine. The 2018 NHIS included questions to ascertain herpes zoster vaccination by type of vaccine (ZVL versus RZV), number of vaccine doses received, and timing of vaccine receipt (*13*).

Results from this study indicated that recently recommended RZV coverage (≥1 dose) was 2.4% among adults aged ≥50 years. ACIP recommended 2 doses of RZV to adults aged ≥50 years (*43*). This study showed that in 2018, RZV coverage (≥2 dose) was 0.6% among adults aged ≥50 years. More RZV doses were distributed in the third and fourth quarters (64%) in 2018 compared with the first two quarters (36%) (CDC unpublished data, 2018), and uneven distribution of this new vaccine could have had an impact on vaccine receipt, estimation of vaccination coverage, and series completion. The results from this study provides first-year RZV coverage following the 2017 ACIP recommendation and a baseline for assessing changes in herpes zoster vaccination coverage following introduction of RZV. Monitoring RZV vaccine use is important for developing strategies to improve coverage for this newly recommended vaccine.

Overall, herpes zoster vaccination coverage for adults aged ≥60 years was 34.5% in 2018, similar to the 2017 estimate and 4.5 percentage points above the *Healthy People 2020* target of 30% (*10*). Although the *Healthy People 2020* target was achieved, approximately 65% of adults recommended to receive this vaccine remain unprotected. Barriers that might have constrained overall herpes zoster vaccination uptake include shortages of herpes zoster vaccines (e.g., there was a ZVL shortage in 2011 and a RZV shortage in 2018) as well as financial and logistic challenges (*44*,*45*). The high cost for providers to purchase a supply and high out-of-pocket costs for patients are well-documented barriers (*46*,*47*). For ZVL, challenges existed to stocking the vaccine (which requires freezer storage), and for ZVL and RZV, variation in out-of-pocket payments for some Medicare Part D beneficiaries existed depending on their specific plan (*46*,*47*). RZV must be stored in a refrigerator (but should not be frozen) and administered immediately after reconstitution or stored in a refrigerator and used within 6 hours. Studies showed that provider recommendation was a strong predictor for vaccination (*48*,*49*). Health care providers should routinely assess patients’ vaccination status and strongly recommend needed vaccines to adults (*48*,*49*).

### Tetanus Toxoid–Containing Vaccination

ACIP updated the adult Tdap vaccination recommendation to include all adults aged ≥19 years who have not yet received a dose of Tdap, including those aged ≥65 years, in 2012 (*50*). Tdap should be administered regardless of interval since the last Td shot. A single dose of Tdap is particularly important for adults who have or who anticipate having close contact with an infant aged <1 year (e.g., parents, grandparents, childcare providers, and HCP) to reduce risk for transmitting pertussis to infants too young to be vaccinated, who are at the greatest risk for severe pertussis including hospitalization and death. Overall, Tdap coverage has remained low for all age groups and among adults living with an infant aged <1 year. In 2018, although there was no increase compared with the 2017 estimate, the trend test found that Tdap coverage increased significantly from 2010 to 2018. Health care providers should not miss an opportunity to vaccinate adults aged ≥19 years who have not received Tdap previously.

Vaccination also offers the best protection against pertussis infection in HCP (*51*–*53*). In 2006, ACIP recommended that HCP aged 19–64 years receive a single dose of Tdap to reduce the risk for transmission of pertussis in health care settings (*52*). In 2010, ACIP updated HCP recommendations indicating that all HCP, regardless of age, should receive a single dose of Tdap as soon as feasible if they had not previously received Tdap (*24*). Vaccinating HCP with Tdap can be a cost-effective strategy to prevent outbreaks in health care settings (*51*–*53*). However, as of 2018, Tdap vaccination coverage among HCP is suboptimal (55.8%).

Tdap vaccination coverage among HCP was lower compared with influenza and hepatitis B vaccination coverage among HCP. Influenza and hepatitis B vaccines are two other vaccines recommended for HCP in the United States (*24*,*54*). Influenza (2017–18 season) and hepatitis B (2018) vaccination coverage among HCP was 71.8% and 67.2%, respectively. Coverage among HCP with direct patient care was 72.6% and 75.3%, respectively. However, influenza and hepatitis B vaccination have been recommended for HCP since 1984 and 1982, respectively, compared with Tdap, which has been recommended for HCP only since 2006 (*52*,*54*,*55*). Other factors, such as perceived risk, employer requirements, and targeted vaccination campaigns, also might contribute to higher influenza and hepatitis B vaccination among HCP (*54*–*57*). Since Tdap vaccination coverage was first assessed in the United States in 2008 (*52*), Tdap coverage among HCP has increased from 15.9% in 2008 (*58*) to 55.8% in 2018. Continued monitoring of Tdap vaccination among HCP is useful for evaluating vaccination campaigns and planning and to control pertussis among HCP and their contacts.

### Hepatitis A Vaccination

Hepatitis A is an acute infection that can result in mild illness or be severe enough to result in hospitalization or, rarely, in death. Incidence rates decreased by approximately 95% from 1995 to 2011, then increased by 140% from 2011 to 2017 (*59*). Incidence rates in the United States have been influenced by occasional outbreaks, often linked to imported food, and among nonimmune persons experiencing homelessness (*60*). Although the average number of annual hepatitis A virus (HAV) infections reported to CDC in recent years has declined substantially compared with 2000, fluctuations have occurred during the preceding 20 years because of large outbreaks. After a long downward trend, the first increase between 2012 and 2013 (1,562 and 1,781 reported cases, respectively) was because of a large multistate outbreak associated with pomegranate arils imported from Turkey (*61*). From 2015 to 2016, reported cases again increased by 44.4% from 1,390 to 2,007 cases. The 2016 increase was caused by two hepatitis A outbreaks, each of which was linked to imported foods. Increases might be expected because of ongoing outbreaks reported to CDC among persons who use drugs, persons experiencing homelessness (*62*), and men who have sex with men (*63*). Men who have sex with men should be vaccinated against hepatitis A and hepatitis B and tested for hepatitis B. Optimal use of vaccination can substantially reduce the hepatitis A disease burden (*64*). One study found that among U.S.-born adults aged ≥20 years, HAV susceptibility prevalence (total antibody to HAV negative) was 74.1% during 2007–2016, indicating that HAV immunity levels among adults was low (*65*). In 1995, the first hepatitis A vaccine became available in the United States. ACIP recommended hepatitis A vaccination of international travelers, men who have sex with men, persons who use injection and noninjection drugs (i.e., all those who use illegal drugs), persons who have occupational risk for exposure, persons who anticipate close personal contact with an international adoptee, persons experiencing homelessness, persons infected with HIV, persons with chronic liver disease, persons living in group settings for those with developmental disabilities, persons who are incarcerated, pregnant women who are identified to be at risk for HAV infection during pregnancy, and adults aged >40 years (*66*).

Information on hepatitis A vaccination was available for the adult general population and selected populations for whom hepatitis A vaccination specifically is indicated (only for those with foreign travel to areas of high or intermediate endemicity and those with chronic liver disease). Although hepatitis A vaccination of adult travelers was higher during 2010*–*2018 than among adult nontravelers, overall hepatitis A vaccination among travelers aged ≥19 years and adults aged ≥19 years with chronic liver disease has remained low (as of 2018, 18.9% and 15.8%, respectively). HCP are encouraged to assess the needs of their patients for hepatitis A vaccine and offer it when appropriate. To further improve hepatitis A vaccination coverage and reduce the burden of hepatitis A infection in the United States, HCP are encouraged to adopt strategies to identify candidates for hepatitis A vaccination (e.g., implementing standing orders in electronic medical records, collocating vaccination at homeless shelters and syringe service programs, and offering vaccine to residents and staff of long-term care centers), and to ensure that traveling adults and all adults at increased risk for hepatitis A infection or seeking protection from hepatitis A infection are offered hepatitis A vaccine (*33*,*34*,*64*,*66*–*68*). Travelers, especially healthy travelers with no physician visit, should see their doctor to discuss their travel-related vaccinations and other preventive care services. CDC recommends that international travelers should schedule a visit to a primary doctor or a travel medicine provider 4–6 weeks before their trip (*67*–*69*).

### Hepatitis B Vaccination

ACIP has recommended a 3-dose hepatitis B vaccine series since 1982 for HCP (*70*,*71*), since 1991 for travelers to or persons working in countries with high or intermediate hepatitis B endemicity (*72*), and since 2011 for unvaccinated adults with diabetes aged 19–59 years. In addition, vaccine can be administered to unvaccinated adults with diabetes aged ≥60 years at the discretion of their HCP (*73*,*74*). Despite these longstanding recommendations for hepatitis B vaccination, coverage remained low in 2018. Furthermore, overall hepatitis B vaccination among travelers and adults with chronic liver disease has remained low, although hepatitis B vaccination among travelers was higher in 2018 and preceding years than among nontravelers.

Several factors might contribute to low hepatitis B vaccination among travelers to countries where hepatitis B virus is endemic. Many travelers to international destinations might omit seeking travel health advice because of lack of awareness of the risk for travel-associated infection and travel-related vaccination recommendations (*75*–*77*). Some travelers (e.g., business travelers, journalists, and relief workers) might be notified of travel on short notice and have little time for vaccination before departure, even though these travelers should be vaccinated in expectation of travel to hepatitis B virus–endemic areas to protect themselves (*75*–*77*). Travelers might believe that travel of short duration, to resorts or on tours, will pose little risk for travel-related diseases (*78*–*81*). HCP are encouraged to adopt strategies to identify candidates for hepatitis B vaccination and to ensure that traveling adults, all adults at increased risk for hepatitis B infection, or those seeking protection from hepatitis B infection are offered hepatitis B vaccine (*75*–*81*). Travelers to a country of high or intermediate hepatitis B endemicity are encouraged to schedule a visit with their doctor or a travel medicine provider 4–6 weeks before travel to discuss the need for travel-related vaccinations (*75*–*77*).

In addition, during 2010–2018, estimates of hepatitis B vaccination among HCP did not improve, ranging from 61% to 67%, well below the *Healthy People 2020* target of 90% (*10*). Hepatitis B vaccination coverage among HCP with direct patient care was higher (75%), although still below the *Healthy People 2020* target (*10*). Before hepatitis B vaccination was widely implemented, hepatitis B virus (HBV) infection was recognized as a common occupational risk among HCP (*82*,*83*). Routine hepatitis B vaccination of HCP and the use of standard precautions have resulted in a 98% decline in HBV infections among HCP from 1983 through 2010 (*84*). The Occupational Safety and Health Administration mandates that employers offer hepatitis B vaccination to all personnel who have occupational risk and that postexposure prophylaxis be available following an exposure (*74*,*84*,*85*). Continued efforts are needed to increase hepatitis B vaccination coverage among unvaccinated HCP to protect workers and patients (*86*).

### HPV Vaccination

HPV is the most common sexually transmitted infection in men and women in the United States (*87*–*91*). Vaccination can prevent HPV infection and associated diseases including genital warts, precancerous lesions, anogenital cancers, and oropharynx cancer (*87*). In 2006, quadrivalent HPV vaccine was recommended by ACIP for use in females aged 11 or 12 years and for those aged 13–26 years who had not been vaccinated previously or who had not completed the 3-dose series (*87*). In 2009, ACIP provided guidance that the quadrivalent vaccine could be given to males aged 9–26 years (a permissive recommendation) (*92*,*93*). In 2011, ACIP recommended routine use of HPV vaccine among males aged 11 or 12 years and for those aged 13–21 years who had not been vaccinated previously or who had not completed the 3-dose series and a permissive recommendation for males aged 22–26 years (*91*,*94*). In 2015, after 9-valent HPV vaccine was licensed, ACIP recommended any of the three licensed HPV vaccines (quadrivalent, bivalent, or 9-valent) for females and quadrivalent or 9-valent vaccine for males among the same age groups previously recommended (*95*). In 2016, ACIP recommended a 2-dose schedule for HPV vaccination of females and males initiating their vaccination before age 15 years (*96*). In 2019, ACIP updated recommendations on HPV catch-up vaccination for U.S. adults to include all persons through age 26 years (*97*). For adults aged 27–45 years, shared clinical decision-making about HPV vaccination is recommended because certain persons who are not adequately vaccinated might benefit (*97*).

Although receipt of at least 1 dose of HPV vaccine increased from 20.7% in 2010 to 52.8% in 2018 for females aged 19–26 years, and from 2.1% in 2011 to 26.3% in 2018 among males aged 19–26 years, as of 2018, coverage has remained low, and many young adult females (47.2%) and males (73.7%) remain unvaccinated and vulnerable to cancers that safe, effective HPV vaccines can prevent. Findings on age at first dose of HPV vaccination of adults indicated that most female and male respondents in the 2018 NHIS reported receiving the first dose of HPV vaccine at age ≥13 years. In 2018, approximately 12% of females and 15% of males aged 19–26 years not vaccinated at age ≤18 years reported receiving the first dose of HPV vaccine as a catch-up dose at age 19–26 years. Since HPV vaccine licensure, multiple cohorts of unvaccinated adolescents and young adults have accumulated. For example, in the 2018 National Immunization Survey–Teen (*98*), provider-reported vaccination histories indicated that 23.7% of females and 35.5% of males aged 17 years were unvaccinated (having not received at least one HPV vaccine dose) (*98*). These estimates reflect the current pool of females and males who could benefit from catch-up vaccination and the number of unprotected older adolescents adding to that pool annually, indicating the importance of catch-up vaccination among young adults.

HCP recommendations for vaccination are strongly associated with a patient’s receipt of vaccines (*34*,*99*–*103*). Findings from one report indicate that among male adolescents with a HCP recommendation, HPV coverage was approximately two times higher than that among those without a provider recommendation (68.8% versus 35.4%) (*48*). The same report found that provider recommendation was associated with higher HPV vaccination coverage in most states (*48*). Another study found that HPV vaccination coverage among female adolescents (≥1 dose) was 58.3% among those with a provider recommendation compared with only 20.7% among those without a provider recommendation (*104*). Other research has indicated that recommendations from providers increase parental acceptance of vaccination of their children and that parents change their minds about delaying and refusing vaccines because of information or assurances from HCP (*105*,*106*). HCP conversations with parents can be an important pathway to achieving higher HPV vaccination coverage of female adolescents, including talking to parents about the HPV vaccine, giving parents time to discuss the vaccine, and making a strong recommendation for HPV vaccination (*107*). However, in 2016, up to 35% of parents of adolescents reported not receiving a provider recommendation for the vaccine (*48*). Increasing HPV vaccination could lead to greater decreases in HPV-attributable diseases in the United States. Continued efforts are needed to improve coverage among members of the primary target group for HPV vaccine (girls and boys aged 11–12 years) and among all racial and ethnic groups. As more adolescents are vaccinated at the target age group and age into the adult population monitored in NHIS, vaccine coverage estimates are expected to increase. To reduce the amount of time needed to achieve population-level impacts of vaccination, such as reduction in HPV-associated cancer incidence, efforts are also needed to improve catch-up vaccination through age 26 years among those who have not started or completed their vaccination (*4*,*97*). Providers should assess vaccination status at clinical encounters, educate persons about the diseases that can be prevented by vaccines, and strongly recommend indicated vaccines (*34*,*99*,*108*).

### Trends in Adult Vaccination Coverage

Although the point estimates for each year varied by only a few percentage points, linear trend tests indicated that during 2010–2018, vaccination coverage increased for influenza (among adults aged ≥19 years overall and those with high-risk conditions), pneumococcal (among those aged 19–64 years at increased risk and adults aged ≥65 years), herpes zoster (among adults aged ≥60 years), Tdap (among adults aged ≥19 years), hepatitis A (among adults aged ≥19 years and travelers or nontravelers aged ≥19 years), hepatitis B (among adults aged ≥19 years), and HPV (among women aged 19–26 years) vaccines, and during 2011–2018 for HPV vaccine (among men aged 19–26 years). Although these increases were small, collectively they might have resulted in meaningful reductions in disease among adults (*31*). Hepatitis B vaccination coverage plateaued among adults aged ≥19 years with chronic liver conditions and travelers or nontravelers aged ≥19 years.

### Racial and Ethnic Differences in Vaccination

In 2018, racial/ethnic differences in vaccination coverage persisted for all seven vaccines assessed in this report. Generally, higher coverage was observed for White adults compared with most other groups. Black, Hispanic, and Asian adults had lower vaccination coverage than Whites for all vaccines routinely recommended for adults, with a few exceptions. Among HCP, there were differences for influenza, Tdap, and hepatitis B vaccination, with White HCP generally having higher vaccination coverage compared with Black and Hispanic HCP.

The findings provided in this report are consistent with previous studies (*4*,*109*). Although studies indicate that racial and ethnic disparities in childhood vaccination have been reduced substantially or not observed for certain vaccinations (*98*,*110*,*111*), racial and ethnic disparities in adult vaccination persist (*4*,*28*,*29*,*111*–*118*). School entry vaccination requirements and the Vaccines for Children program, which provides vaccines to children who might otherwise be unable to afford them, might contribute to reduced racial and ethnic disparities in vaccination coverage for children (*119*–*121*). Multiple factors contribute to racial and ethnic differences in adult vaccination, including differences in attitudes toward vaccination and preventive care, propensity to seek and accept vaccination, variations in the likelihood that providers recommend vaccination, differences in quality of care received by racial and ethnic populations, and differences in concerns about vaccination including vaccine safety (*111*–*118*). In addition, non-Hispanic Black and Hispanic adults are more likely to be uninsured (*122*). Lack of medical insurance has been an important predictor of low adult vaccination uptake (*4*,*117*,*123*). Another factor that might contribute to coverage disparities is differential awareness of vaccines. Studies have shown that awareness of Tdap, herpes zoster, and HPV vaccines was significantly lower among racial and ethnic minorities compared with non-Hispanic Whites (*57*,*102*,*123*,*124*). Older Black adults report more negative attitudes toward influenza vaccination than White adults (*113*); however, studies of standardized offering of influenza and pneumococcal vaccines have demonstrated reductions in racial and ethnic coverage disparities (*125*,*126*). Using a combination of patient tracking, vaccination reminders for providers and patients, and patient outreach and assistance also reduces racial/ethnic vaccination differences (*103*). Incorporating standards for adult vaccination practices, which include routinely assessing vaccination needs during all clinical encounters, providing a strong recommendation for vaccination to patients with indications, and then offering vaccination at the visit (*34*) or referring patients for vaccination elsewhere, can reduce vaccination disparities.

### Access-to-Care Characteristics and Adult Vaccination Coverage

Consistent with a previous report (*127*), in this study having health insurance was generally associated with a greater likelihood of having received recommended vaccinations, even after controlling for demographic and access-to-care variables. For many of the vaccines, coverage was greater among adults with private health insurance compared with those reporting public health insurance, but this finding was not consistent for all vaccines and age groups. The factors contributing to vaccination levels by type of health insurance are not well understood. Health insurance coverage, although beneficial in improving access to health care services, might not be sufficient in itself to achieve optimal adult vaccination. In this report, even among adults who had health insurance and ≥10 physician contacts during the preceding year, up to 87.5% reported not receiving one or more recommended vaccines. Provider attitudes toward adult vaccination, practice patterns that do not routinely incorporate assessments for vaccines for adults, and other barriers to vaccination might determine whether patients are offered and receive vaccines (*127*–*133*).

Generally, persons with a usual place for health care were more likely to report having received recommended vaccinations than those who did not have a usual place for health care, regardless of whether they had health insurance, and vaccination coverage generally increased as the number of physician contacts increased. Having a usual place for health care and routine physician contact can provide important opportunities for providers to educate their patients about vaccine-preventable diseases and to recommend and offer vaccination (*102*,*109*,*117*,*134*). However, a recent study showed that overall, among adults with a doctor visit, only 57.0% received a provider recommendation for influenza vaccination (*49*). Patients usually trust the opinions of HCP regarding vaccination more so than opinions from others (*34*,*135*). However, only 32% of family physicians and 29% of internists assess their adult patients’ vaccination status at every visit (*135*).

### Adult Vaccination Coverage by Nativity, Years Living in the United States, and Citizenship

Results from this study indicated that adult vaccination coverage was generally lower among foreign-born compared with U.S.-born persons. Vaccination coverage for foreign-born persons differed by time lived in the United States. A previous study showed that vaccination was also associated with language used for interview, race/ethnicity, and birth country/region (*136*). Among foreign-born persons, vaccination coverage was generally lower among those who were not U.S. citizens, those interviewed in a language other than English, and non-Hispanic Blacks or Hispanics. Hispanic foreign-born adults had the lowest coverage for several vaccines. This finding is particularly relevant because foreign-born persons from Latin America account for more than half of all foreign-born adults in the United States (*137*–*139*). Vaccination coverage and immunization schedules are different in many countries compared with the United States and vary by country and even by regions within countries (*136*,*140*,*141*). Although immigrant visa applicants and refugees destined for permanent resettlement in the United States are subject to ACIP-recommended vaccination requirements, the differences between the United States and other countries in the schedules of routine vaccinations among adults might contribute to differences in the coverage levels of the vaccines studied. Public policymakers, vaccination programs, and HCP should consider foreign-born populations in their public health assessment, evaluation, and outreach programs that target disadvantaged groups (*142*).

### Improving Adult Vaccination Coverage

Studies indicate that a strong HCP recommendation is closely associated with patient vaccination (*48*,*49*,*128*). Standards for Adult Immunization (the Standards) was published for implementing ACIP recommendations and outlining approaches for improving adult vaccination coverage (*33*,*34*). Wider adoption of the Standards (i.e., assessing vaccination status at each adult patient visit, issuing strong recommendations for indicated vaccines, offering vaccines or referring patients to other providers for vaccination, and recording vaccinations received in the Immunization Information System [IIS]) (*33*,*34*) will help improve vaccine coverage. Research suggests medical specialists are less likely than primary care clinicians to assess for, recommend, stock, or refer patients for needed vaccines (*143*). Because patients with conditions placing them at increased risk for infection are likely to receive care from specialists, these encounters might represent missed opportunities for vaccination and could be addressed by consistent implementation of the Standards by these providers. Among the challenges clinicians face in assessing the need for vaccination is availability of a complete and accurate vaccination history along with access at the point of care to the most current vaccination recommendations. Enhancing provider access to IIS could help improve vaccination coverage because IIS can provide consolidated immunization histories for use by a vaccination provider in determining appropriate client vaccinations (*144*). Nationwide adoption of electronic health records, many of which have the capacity for patient-centered clinical decision support, also offer opportunities for improving adult vaccination coverage (*4*).

Standardized offering of vaccines reduces but does not eliminate racial/ethnic differences in coverage (*15*). Although programmatic initiatives designed to improve adult vaccine coverage overall might have a positive effect on these disparities (*125*), their persistence in the face of years of such intervention suggests that novel and systematic approaches are required. More information on contributors to such disparities will be necessary to inform the design of meaningful interventions to further improve vaccination among adult populations.

## Limitations

The findings in this report are subject to at least eight limitations. First, the NHIS sample excludes persons in the military and those residing in institutions, which might result in underestimation or overestimation of overall U.S. vaccination coverage levels. Second, reported vaccination status was not validated with medical records. However, adult self-reported vaccination status has been shown to be ≥70% sensitive in one or more studies for pneumococcal, tetanus toxoid–containing, herpes zoster, and hepatitis B vaccines and ≥70% specific in one or more studies for all except tetanus and hepatitis B vaccination (*145*–*148*). Third, demographic and other reported characteristics (e.g., insurance status, usual source, and frequency of health care) also were not validated. Fourth, adults might not be able to recall accurately vaccines received as infants or adolescents and as a result, coverage levels for hepatitis A, hepatitis B, HPV, and Tdap vaccination might be substantially underestimated. Age at receiving the first dose of HPV might be subject to recall bias. Additional studies are needed to determine accuracy of recall for vaccinations that adults might have received as children or adolescents. Fifth, the response rate was 53.1%. Nonresponse bias can result if respondents and nonrespondents differ in their vaccination rates, and if survey weighting does not fully correct for this. Sixth, the Tdap estimate is subject to considerable uncertainty. Respondents who reported a tetanus vaccination during the preceding 10 years but were unable to say whether Td or Tdap was used for their most recent shot were excluded from estimations of Tdap coverage, creating a potential for bias. Sensitivity calculations were conducted to assess the magnitude of potential bias. Depending on what proportion of excluded respondents actually received Tdap, coverage with Tdap could fall within the ranges of 19.9%–56.1% for adults aged ≥19 years, 21.5%–57.2% for adults aged 19–64 years, and 13.8%–51.6% for adults aged ≥65 years. Seventh, a recombinant, adjuvanted hepatitis B vaccine requiring 2 doses 4 weeks apart was licensed in 2017 and recommended by ACIP in February 2018 as an option for previously unvaccinated or incompletely vaccinated persons, including adults aged ≥18 years who have a specific risk or lack a risk factor but want protection (*149*). The 2018 NHIS did not include questions to ascertain the type of hepatitis B vaccine used or number of doses received by type of vaccine. Finally, the prevalence of selected behavioral characteristics in populations, including the use of preventive health services, vaccine safety concerns, state laws and immunization intervention programs and cultural, religious, and other factors might affect vaccination coverage. Although NHIS collects information on use of other preventive health services, this information was not included in this analysis. NHIS did not collect information on the other factors.

## Conclusion

In 2018, coverage for the composite adult vaccination quality measure was low in all age groups. Individual adult vaccination coverage in 2018 remained similar to that in 2017, but modest gains occurred in vaccination coverage for certain vaccines. Racial/ethnic vaccination differences persisted for routinely recommended adult vaccines. Trend tests indicated coverage increases from 2010 to 2018 for most vaccines assessed, although overall coverage remained low. Assessing the associations between vaccination and sociodemographic and other factors is important for understanding factors that contribute to low coverage rates and to differences in vaccination, and for implementing strategies to improve vaccination coverage. Awareness of the need for vaccines for adults is low among the general population, and adult patients rely on provider recommendations for vaccination (*49*,*57*,*128*,*130*). The adult immunization schedule (*8*), updated annually, provides current recommendations for vaccinating adults and a resource for persons who provide health care services for adults in various settings. Achieving improvement in overall vaccination coverage, while reducing racial/ethnic vaccination disparities, will require action at multiple levels of the health care system. The composite adult vaccination quality measure might help facilitate evaluation of interventions designed to reduce racial/ethnic vaccination differences and increase the number of U.S. adults who are fully protected against vaccine-preventable diseases. In addition, the COVID-19 pandemic might have affected adult vaccination coverage in 2020 because fewer adults might have visited HCP where vaccinations are administered. A recent study identified declines in routine pediatric vaccine ordering and doses administered (*150*). Disruptions to primary care also have been reported (*151*). Routine vaccination is an essential preventive care service for children, adolescents, and adults (including pregnant women) that should not be delayed because of the COVID-19 pandemic. Guidance on resuming safe vaccination of adults is available (*152*). Many of the long-established public health actions to increase vaccination coverage among adults can be used for both routine vaccinations and for a COVID-19 vaccine. Because of COVID-19–related reductions in persons accessing vaccination services, it is important to assess the vaccination status of all patients at each visit to avoid missed opportunities for vaccination and ensure timely vaccine catch-up. All vaccines due or overdue should be administered according to the recommended CDC immunization schedules during that visit, unless a specific contraindication exists, to provide protection as soon as possible and minimize the number of health care visits needed to complete vaccination.

## Appendix Ascertainment of Vaccination Status

Influenza vaccination: Respondents were asked if they had received an influenza shot or nasal spray during the preceding 12 months and, if so, in which month and year.

Pneumococcal vaccination: Respondents were asked if they had ever had a pneumonia shot, and if yes, how many doses were received. There were no questions in the 2018 National Health Interview Survey (NHIS) to ascertain pneumococcal vaccination by type of vaccine (23-valent pneumococcal polysaccharide vaccine or 13-valent pneumococcal conjugate vaccine).

Tetanus vaccination: Before the 2017 NHIS, respondents were asked if they had received a tetanus shot during the preceding 10 years. Respondents who had received a tetanus shot during the preceding 10 years were asked if their most recent shot was received in 2005 or later. Respondents who had received a tetanus shot since 2005 were asked if they were told that their most recent tetanus shot included the pertussis or whooping cough vaccine. Starting from the 2017 NHIS, respondents were asked if they had received a tetanus shot in the past 10 years. Respondents who had received a tetanus shot in the past 10 years were asked if they were told that their most recent tetanus shot included the pertussis or whooping cough vaccine.

Hepatitis A vaccination: Respondents were asked if they had ever received the hepatitis A vaccine and, if yes, how many doses were received.

Hepatitis B vaccination: Respondents were asked if they had ever received the hepatitis B vaccine and, if yes, whether they had received ≥3 doses or <3 doses.

Herpes zoster vaccination: Before the 2018 NHIS, respondents were asked if they had ever received a shingles vaccine. Starting with the 2018 NHIS, respondents were asked if they had ever received a shingles vaccine and, if yes, what type of vaccine received (zoster vaccine live or recombinant zoster vaccine), number of vaccine doses received, and timing of vaccine receipt.

Human papillomavirus vaccination (HPV): Respondents were asked if they had ever received an HPV shot or cervical cancer vaccine and, if yes, how many doses were received and age at the first dose.

**Classification as Health Care Personnel**

Adults were classified as health care personnel if they reported that they volunteer or work in a hospital, medical clinic, doctor’s office, dentist’s office, nursing home, or some other health care facility, including part-time and unpaid work in a health care facility, including emergency responders and public safety personnel, as well as professional nursing care provided in the home. If yes, respondents were asked whether they provide direct patient care (physical or hands-on contact with patients) as part of their work.

**Ascertainment of Travel Status**

Respondents were asked whether they had ever traveled outside the United States to countries other than Europe, Japan, Australia, New Zealand, or Canada since 1995.

**Ascertainment of Health Insurance Status**

Adults were considered insured if they reported having public health insurance coverage (Medicare, Medicaid, military health care [TRICARE/VA/CHAMP-VA], Indian Health Service, state-sponsored health plan, or other government program insurance) or private health insurance coverage.

**Ascertainment of Having a Usual Place for Health Care**

Respondents were asked if there is a place to which they usually go when sick or need advice on their health. Respondents answering “yes” were defined as having a usual place for health care.
